# Potential use of beneficial fungal microorganisms and C-phycocyanin extract for enhancing seed germination, seedling growth and biochemical traits of *Solanum lycopersicum* L.

**DOI:** 10.1186/s12866-022-02509-x

**Published:** 2022-04-21

**Authors:** Rabab A. Metwally, Reda E. Abdelhameed, Shereen A. Soliman, Asmaa H. Al-Badwy

**Affiliations:** Botany and Microbiology Department, Faculty of Science, Zagazig University, Zagazig, 44519 Egypt

**Keywords:** Biostimulants, *Trichoderma* sp., *Beauveria*, C-phycocyanin, Flavonoid content, Hydrolytic enzymes, Tomato

## Abstract

**Background:**

Biopriming as a new technique of seed treatment involves the application of beneficial microorganisms on the seed surface to stimulate seed germination, plant growth, and protect the seed from soil and seed-borne pathogens. The present investigation was carried out on seed germination, seedling vigor and biochemical traits of one of the most important vegetable crops (Tomato, *Solanum lycopersicum* L.). The treatments comprised viz. T1: Non primed seeds (Control), T2: Hydropriming, T3: Biopriming with C-phycocyanin (C-PC) (*Spirulina platensis* extract), T4: Biopriming with *Trichoderma asperellum*, T5: Biopriming with *T. viride*, T6: Biopriming with *Beauveria bassiana*.

**Results:**

Extraction and purification of C-phycocyanin (C-PC) from the dry *S. platensis* powder using various methods was performed. The purity after dialyses was 0.49 and its ultimate purity (A_620_/A_280_) after ion-exchange chromatography was 4.64. The results on tomato seedlings revealed that the maximum germination percentage (100%), germination index (15.46 and 15.12), seedling length (10.67 cm), seedling dry weight (1.73 and 1.97 mg) and seedling length vigor index (1066.7) were recorded for tomato biopriming with *T. viride*, and *B. bassiana* (T5 and T6). Moreover, the quantitative estimation of total carbohydrates and total free amino acids contents in bioprimed tomato seedlings indicated a significantly higher amount with *T. viride*, followed by those bioprimed with *T. asperellum*, *B. bassiana* and C-PC extract.

**Conclusion:**

Thus, our results indicated that biopriming of tomato seeds with beneficial fungal inoculants and C-PC was very effective. The most operative biostimulants were those bioprimed with *T. viride* and *B. bassiana* compared to other biostimulants (*T. asperellum* and C-PC). Therefore, to ensure sustainable agriculture, this study offers new possibilities for the biopriming application as an alternative and ecological management strategy to chemical treatment and provides a valuable basis for improving seed germination.

## Background

Agricultural practices are continually being modernized to keep up with the ever-changing environment, with the introduction of genetically modified crops, plant growth regulators, fungicides, fertilizers, pesticides and so on. Their advantages, however, come at a price: some are time-consuming and costly to implement, while others are regarded as detrimental to consumer health and the environment in the long run [1, 2]. As a result, scientists must devise strategies that improve agricultural yield while minimizing hazards. Plant growth regulators and biostimulants are gradually becoming the primary research fields among many scientific researchers to improve plant growth and development [3–5]. Additionally, the positive association and interaction of rhizo-competent microbes are frequently employed for plant bio fertilization and stress-induced damage mitigation [6]. A plant biostimulant is a product that encourages plant nutrition processes independently of its nutrient content. The sole purpose of it is the enhancement one or more of the subsequent characteristics of the plant and its rhizosphere such as accessibility of restricted nutrients in soil or rhizosphere, nutrient use performance, tolerance to abiotic stress and quality characters [7, 8]. Moreover, the biostimulant stimulates the response to the environment, such as stress circumstances or phytopathogenic attack [9, 10]. Furthermore, it can as well help plants to grow and develop in a variety of ways from seed germination to maturity, including improving metabolism to improve yield and crop quality, interacting with nutrient assimilation and translocation, facilitating plant defense against adverse conditions, and so on [11]. Also, Rouphael et al. [7] and Calvo et al. [11] stated that the stimulation of germination, seedlings and plant growth as well as crop productivity in response to plant biostimulants has been usually related to the action of signaling bioactive molecules in the primary and secondary metabolisms. To fully understand the biological role of biostimulants, it’s necessary to evaluate the subject plants’ growth stage and pattern, as well as their developmental reactions [1, 3]. As a result, seed germination, being a crucial step in the growth of a new plant, it reflects the plants’ lateral growth pattern, the fitness, survival, persistence and evolutionary potential of plants [12]. Internal and external factors are heavily regulating the seed’s dormancy status and germination potential at this stage.

Microbial biopriming is an adaptive approach for improving a plant’s defensive capacity, resulting in enhanced resistance/stress tolerance and/or a more exacerbated defense response to stress-inducing circumstances before germination [13, 14]. It also effectively reduces the dependence on chemical fungicide for diseases management [6, 15]. In seed biopriming, seeds were coated with a variety of agriculturally significant microorganisms, resulting in quick and consistent seed colonization [14, 16]. However, if seeds are infected with undesired indigenous microorganisms, they may proliferate during priming and may reduce the survivability of beneficial microbes [17], hence disinfecting the seeds before priming is required [15, 16].

The stimulatory consequence of biostimulants, such as algal extracts and plant-growth promoting fungi (PGPF), on seed germination and seedlings growth has been previously documented [14, 18, 19]. A representative, cyanobacteria can be used to make useful plant products such as fertilizers that play an important role in sustainable agriculture, helping to increase soil fertility, crop development, and environmental quality [20, 21]. Vitamins, amino acids, polypeptides, phytohormones (gibberellins, auxins, cytokinins), antioxidants, and substances with antibacterial and antifungal effects can all be found in *Spirulina platensis*, which can be exploited as a rich source of macro, micronutrients and proteins such as phycobiliproteins for plants [20, 22]. One of the most important phycobilliproteins is C-phycocyanin (C-PC; derived from cyanobacteria like *S. platensis*) which has been used as a natural blue dye in commercial applications [23].

Makhaye et al. [24] estimated the influence of algal extract and biostimulant biopriming on the seed germination parameters such as germination percentage and germination index of *Abelmoschus esculentus*. Also, Zhang et al. [19] determined the effect of PGPF (*Trichoderma longibrachiatum*) on wheat seedlings’ growth and improvement under stress, besides examining the role of *T. longibrachiatum* in inducing the resistance at physiological and biochemical levels. *Trichoderma* spp. rhizo-competent’s nature allows it to colonize roots, boost the plant immune system and have been explored as a possible biocontrol agent [25, 26]. Additionally, the colonization of these beneficial fungi promotes plant growth and protects the host plants from abiotic and biotic stressors [5]. Moreover, Russo et al. [27] estimated the improving effect of endophytic fungus *Beauveria bassiana* on seed germination percentage of *Zea mays*.

One of the most important and extensively used vegetable crops is tomato plant (*Solanum lycopersicum* L.), it contains a variety of metabolites that have health and nutritional benefits. It also includes easy-to-maintain diploid genetics, a minimal generation time, and routine transformation technologies [28]. Together these make tomato an excellent model plant for biologists for both basic and applied plant research. Tomato plants hampered with a load of pathogenic seed microflora that led to a number of nurseries such as seed rot and other field diseases. The infected seeds thus used are responsible not only for the poor germination seedlings stand but also for the carryover of pathogens to the field. Moreover, the germination time of tomato seeds is very high as compared to field crops which lead to non-uniform seedling stand and low vigor seedlings [29]. Considering this, to ensure sustainable agriculture, biopriming is regarded as the most effective method of seed protection. Thus, the present study was conducted to extract and purify C-PC from the dry *Spirulina* powder using various methods and to evaluate its biological activity. Furthermore, the comparative effects of biopriming with fungal inoculums such as *T. viride*, *T. asperellum*, *B. bassiana* and C-PC on tomato seedling growth promotion was studied to consider their significant biological applications, in addition, to assess the hydrolytic enzymes activity and other biochemical attributes of tomato seedlings.

## Results and discussion

### Extraction and purification of C-PC

**Fig. 1:**
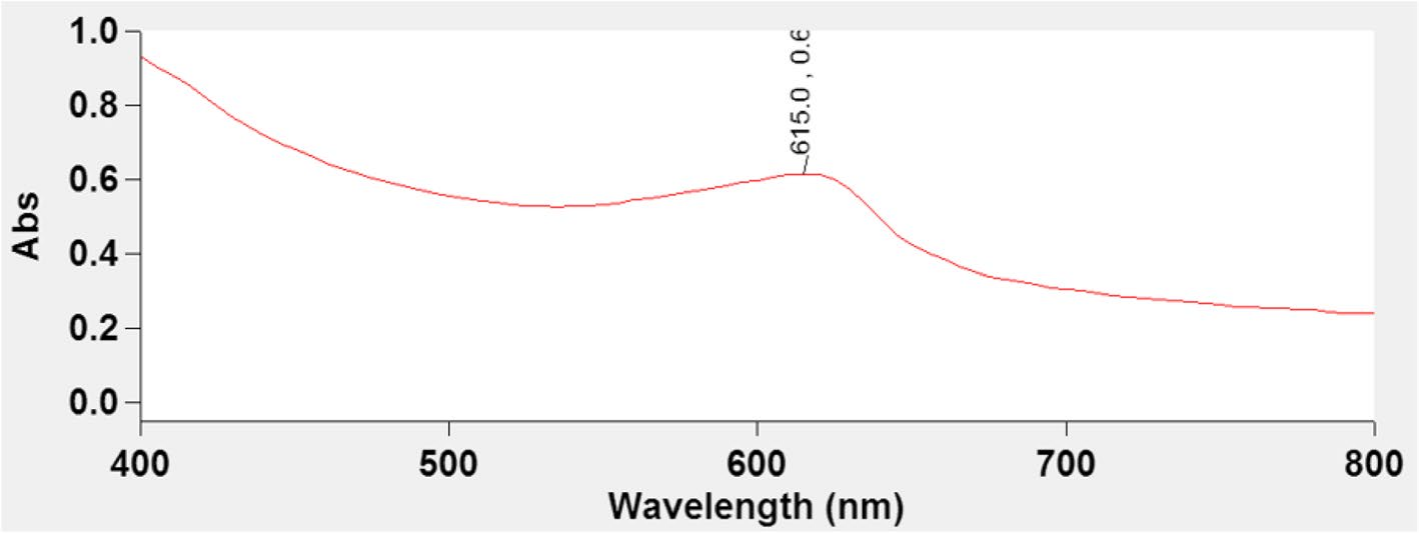
Ultraviolet spectrum of C-PC from *S. platensis*

C-PC was extracted and purified in three steps: crude extract preparation (Step I), dialysis (Step II), and ion-exchange chromatography (Step III). The concentration and purity of C-PC were verified and improved with each purifying process (Table 1). The purity after dialysis was 0.49 and its ultimate purity (A_620_/A_280_) after ion-exchange chromatography was 4.64, where was eluted as a brilliant blue colored solution during the column chromatography. The purity of C-PC was evaluated at each fraction and increased about 9 times in the third fraction (highest purity) more than dialysis (Table 1). The highly pure C-PC’s absorption spectra revealed a strong peak at 615 nm (Fig. 1). Eriksen [30] documented that *Spirulina* is widely implemented as a high-quality protein for C-PC as a cyanobacterial accessory pigment with a variety of agricultural and industrial uses. A variety of publications on the extraction and purification of C-PC from cyanobacterial strains are available [31–33].

**Table 1:** Purity of phycocyanin at each purification step

Step		C-PC (μg ml^**− 1**^)	Purity (A_**620**_/A_**280**_)
**Crude Extract**		85.43	0.26
**Dialysis**		680.84	0.49
**Fractions of Ion Exchange Chromatography**	**2**	59.75	0.64
	**3**	248.72	4.64
	**4**	202.86	1.62
	**5**	91.71	1.35
	**6**	52.80	1.20

Extract purities not only vary per strain, but they are also influenced by the extraction methods used, and further purification techniques are frequently used to improve the purity of the extracts [34]. Safaei et al. [35] employed a four-step purification procedure comprising the adsorption of impurities with chitosan, activated charcoal, ammonium sulfate precipitation, and ion-exchange chromatography, reaching a high purity form of C-PC of 5.26. Furthermore, Schipper et al. [32] discovered that the extraction buffer and cell disruption technique has an impact on the C-PC content and extract purity from *Leptolyngbya* sp. and *Arthrospira platensis* and reported that the cell disruption technique with CaCl_2_ was the best approach for *A. platensis*, while it was the second-best method for *Leptolyngbya* sp. In comparison to other approaches, Diethylaminoethyl (DEAE) column chromatography was considered to be an essential method for purifying C-PC from *S. platensis* according to Moovendhan et al. [33]. However, Seo et al. [36] extracted C-PC from *S. platensis* using a hexane separation method and a high-pressure process.

### FT-IR spectral analysis

**Fig. 2:**
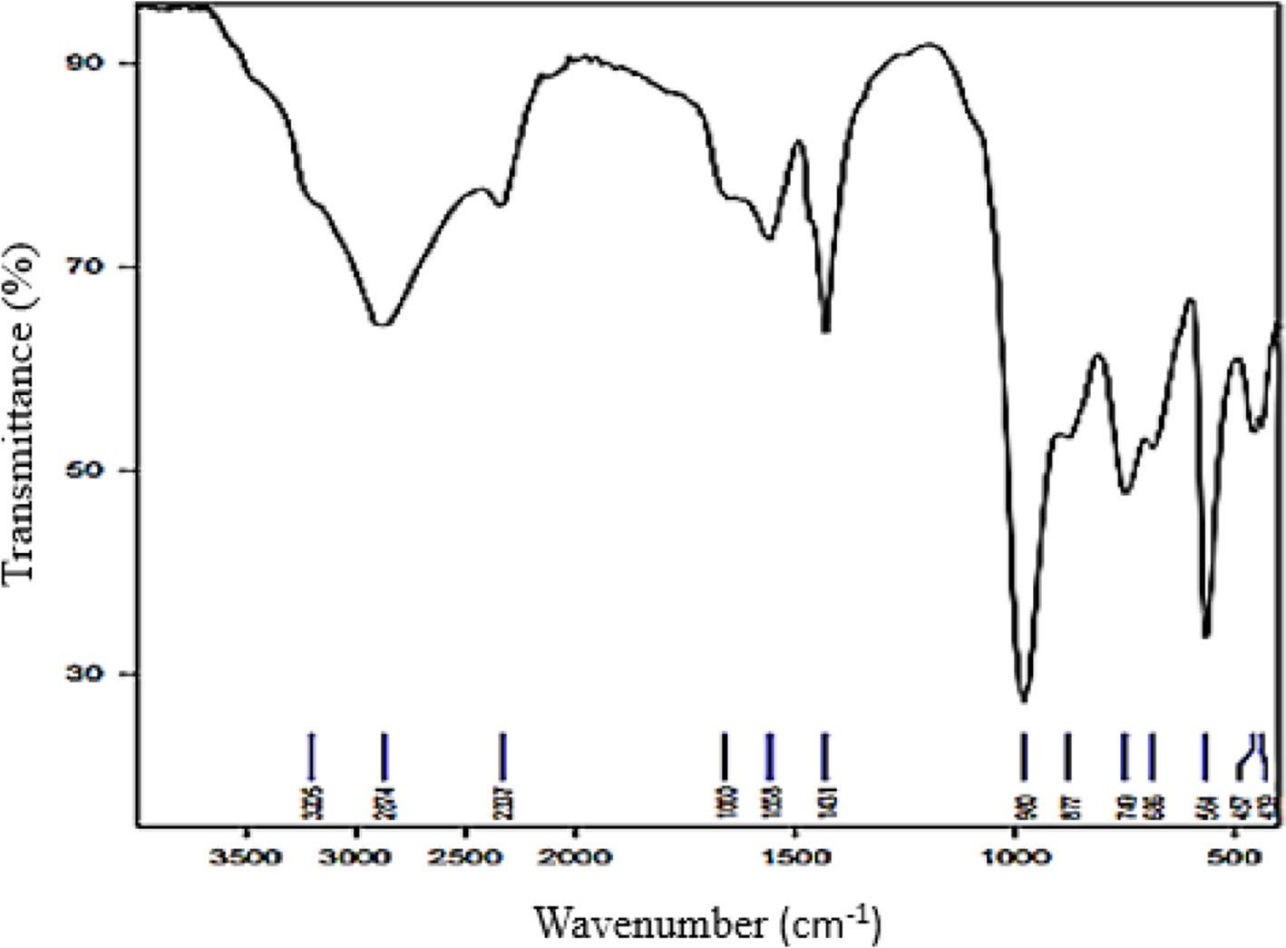
FT-IR spectrum of C-PC

In the current investigation, the C-PC of *S. platensis* revealed functional groups, with peak frequencies of 686.31 and 748.78 cm^− 1^ representing the presence of the C–H bond in the molecule. The CH_2_ bending vibration was identified at 1455 cm^− 1^, and the protein amide II band was detected at 1558 cm^− 1^ (C=O stretching). Furthermore, the presence of carboxylic acids, C = N and N–H bond in the molecule were shown at 2337.10, 1660 and 3205 cm^− 1^ respectively. These functional groups were recorded according to Gokel [37] (Fig. 2). Our findings are similar to those of Moovendhan et al. [33], who studied *S. platensis* C-PC and found functional groups at peak frequencies of 673.86, 794.67, 1456.26, 1539.20, and 2358.94, which are virtually identical to our data. The FT-IR spectrum of *Ulva lactuca* extract revealed C-PC as the most bioactive component, with transmittance maxima at 1652, whereas our results were 1660 cm^− 1^, which is mostly suggested by COO, CO, and conjugated double bonds. These bonds had spectral bands peaking at 2985 cm^− 1^, 2860 cm^− 1^, and 2986 cm^− 1^ [38], which corresponded to 2874 cm^− 1^ in our investigation.

### ^1^H NMR spectral analysis

**Fig. 3:**
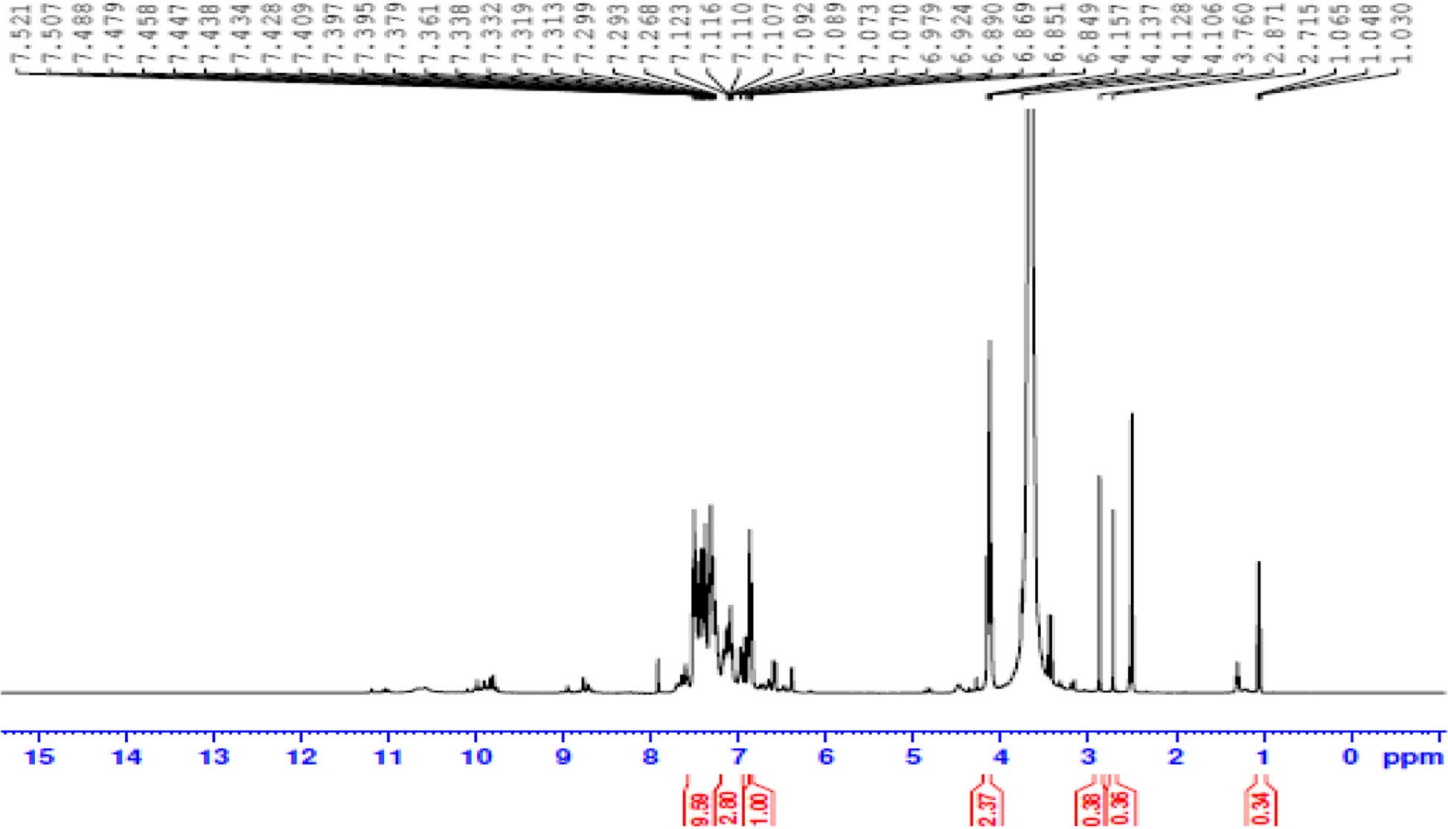
^1^H NMR spectrum of purified C-PC from *S. platensis*

In our investigation, ^1^H NMR spectra were measured at ppm level ranging from 21 to 13 ppm. The chemical shifts of C-PC signal 2.71 (δ), 2.87 (δ) and 3.76 (δ) confirmed the presence of Alkyne (C. C-H) type protons. Chemical shifts 6.86 (δ), 6.89 (δ), 6.92 and 6.98 (δ) proved the presence of an alkene with C-H type protons and N-H. Whereas chemical shift 7.3(δ) confirmed the presence of aromatic with H on the phenyl ring NH. Also, a pyrrolic NH signal was observed at 7.36 (Fig. 3). Wiegand et al. [39] used NMR spectroscopy to investigate the structural characteristics of phycoerythrocyanin peptides from thermophilic cyanobacterium *Mastigocladus laminosus* and *Fischerella* sp., and reported different functional groups with different proton types at various ppm and chemical shifts. Similarly, Moovendhan et al. [33] suggested the presence of 14 chemical shifts (δ) and confirmed the presence of alkyl halide, alkene and aldehyde proton in ^1^HNMR analysis of phycocyanin. With varying concentrations of pigments extracted from *Chattonella verruculosa*, Mangoni et al. [40] observed 19 and 6 chemical changes.

### Germination and growth indices of tomato seedlings after seed biopriming

**Fig. 4:**
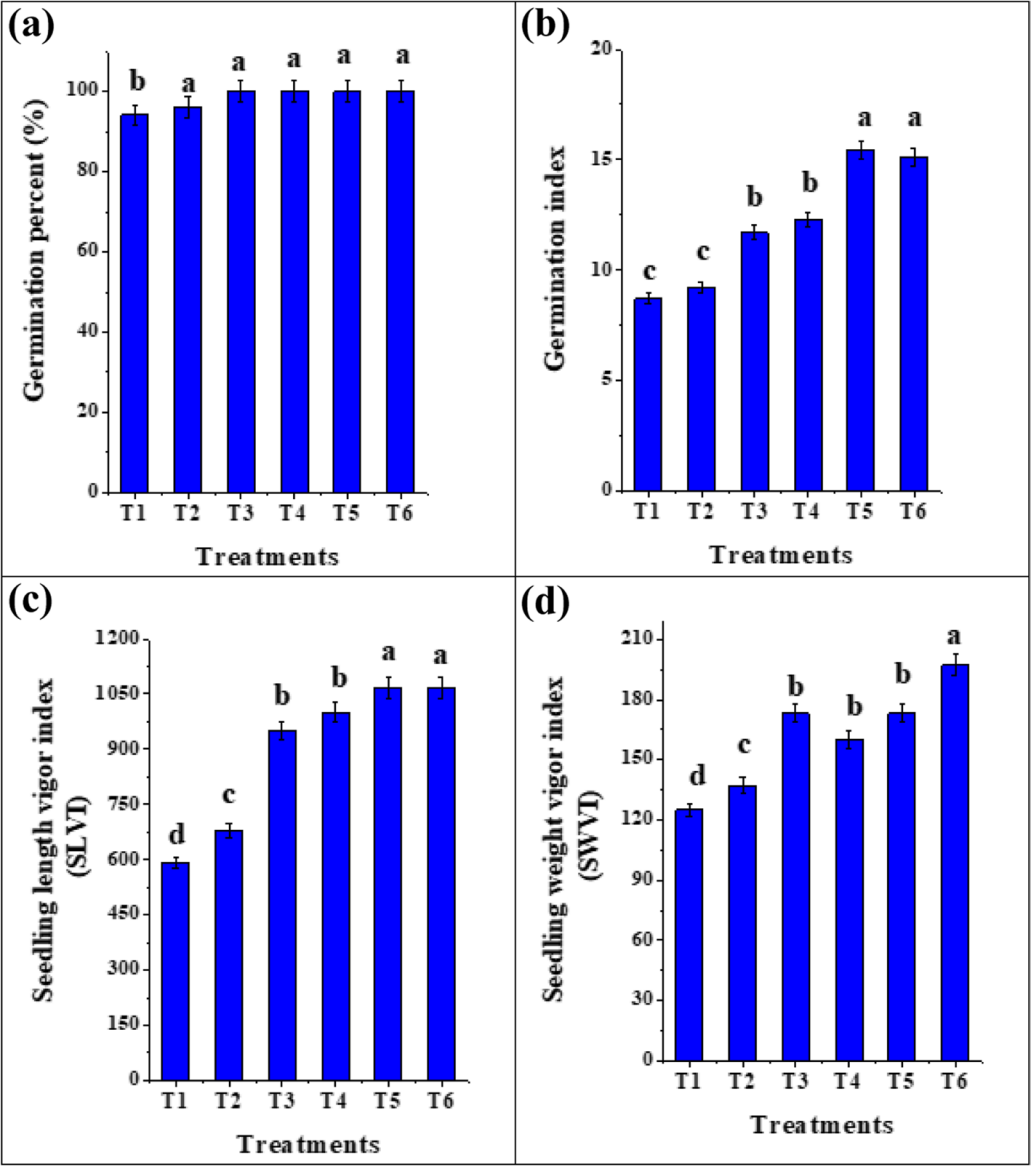
Percentage germination (%), Germination index (GI), seedling length and weight vigor of tomato after seed priming with fungal inoculums and C-PC extract. Tomato seeds were primed for 24 h and germinated for 12 days; (T1) unprimed *S. lycopersicum* seedlings (T2) hydroprimed *S. lycopersicum* seedlings (T3) *S. lycopersicum* bioprimed with C-PC (T4) *S. lycopersicum* bioprimed with *T. asperellum* (T5) *S. lycopersicum* bioprimed with *T. viride* (T6) *S. lycopersicum* bioprimed with *B. bassiana*. *Values are means ± SE (Standard Error). Bars labeled with the different alphabet(s) are significantly different (Duncan’s Multiple Range Test, *p* < 0.05)

**Fig. 5:**
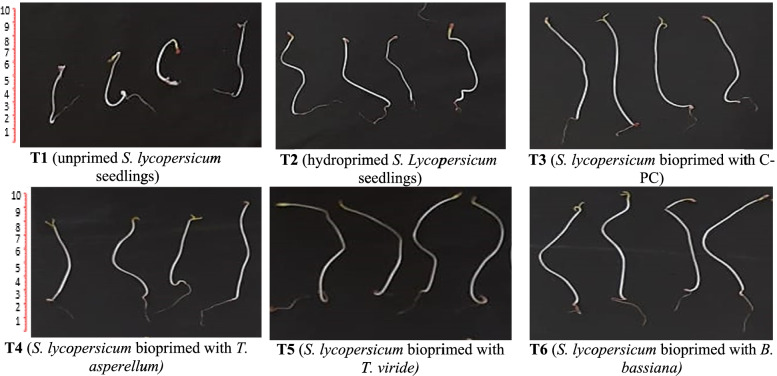
Morphological growth characteristic of unprimed or bioprimed *S. lycopersicum* seedlings. The *S. lycopersicum* bioprimed samples were found to have profuse growth with increased shoot height and radicle length compared to unprimed seedlings

Seed biopriming improves the initial step of plant development by encouraging more uniform seed germination, inducing profound changes in plant characteristics and providing protection before seedling emergence [41]. Figure 4(a-d) depicts the comparative effects of biopriming with fungal inoculums (*T. viride*, *T. asperellum* and *B. bassiana*) and C-PC extract on germination percentage (%), germination index (GI), seedling weight vigor index (SWFI) and seedling length vigor index (SLVI) of tomato seedlings after 12 days of growth. Also, the photograph was taken for *S. lycopersicum* seedlings to show the effect of the priming on the growth indices as compared to unprimed seedlings (Fig. 5). Likewise, the morphological data for tomato seedlings can be observed in Table 2. Concerning germination indices, an increase in SWVI in tomato seedlings primed with *T. asperellum, T. viride* and C-PC (160 and 173) and the maximum records were documented for the tomato seeds bioprimed with *B. bassiana* (197) compared to the unprimed or hydroprimed ones (125 and 137). Moreover, seed biopriming produced a staggering improvement in seedling’s FW, DW, shoot height and radicle length, where the highest values for the seedling FW were recorded for seedlings primed with *T. viride* (49.8 mg) and *T. asperellum* (48.8 mg). In contrast, the unprimed or hydroprimed seeds exhibited significantly (*p* < 0.05) lower records (19.9 and 28.9 mg). Our results are in harmony with Aamir et al. [6] who reported that the biopriming of tomato seeds with *T. erinaceum* caused a profuse growth in morphological attributes. Sánchez-Rodríguez et al. [42] recorded an enhancement in wheat growth colonized by *B. bassiana*. Moreover, Metwally and Al-Amri [43] and Metwally et al. [5] reported an increase in shoot length, root length, shoot and root FW and DW of onion with *T. viride*. Moreover, Russo et al. [27] reported that the percentage of corn seed germination was significantly increased with *B. bassiana*. As, *Trichoderma* spp. and *B. bassiana* are endophytic fungi, enhance plant growth by increasing nutrient uptake and production of plant growth regulators along with induction of secondary root development through indoles acetic acid (IAA), gibberellin, cytokinins and siderophores production [44]. As well, these fungi produce phosphatase as well organic acids which solubilize the inaccessible phosphate to make it available, also help to increase the N_2_ use efficiency in plants [5, 44].

**Table 2:** Different plant growth promotion parameters analyzed on tomato after seed biopriming with C-PC, *T. viride*, *T. asperellum* and *B. bassiana* after 12 days of growth

Treatments	Shoot height (cm)	Radicle length (cm)	Seedling length (cm)	Seedling FW (mg)	Seedling DW (mg)
**Control**	4.67 ± 0.44d	1.63 ± 0.186a	6.30 ± 0.611c	19.97 ± 3.1d	1.33 ± 0.035c
**H**_**2**_**O primed**	5.17 ± 0.088c	1.90 ± 0.208a	7.07 ± 0.296b	28.9 ± 6.8c	1.43 ± 0.037c
**C-PC**	7.50 ± 0.288b	2.00 ± 0.577a	9.50 ± 0.288a	36.1 ± 2.1b	1.73 ± 0.046b
***T. asperellum***	7.83 ± 0.441ab	2.17 ± 0.167a	10.00 ± 0.577a	48.8 ± 2.8a	1.60 ± 0.042b
***T. viride***	9.00 ± 0.577a	1.67 ± 0.333a	10.67 ± 0.666a	49.8 ± 5.9a	1.73 ± 0.046b
***B. bassiana***	8.17 ± 0.167ab	2.50 ± 0.288a	10.67 ± 0.441a	38.7 ± 3.4b	1.97 ± 0.052a

Values are the means ± SE (Standard Error). Means in each column followed by the same alphabet(s) are not significantly different at *p <* 0.05 (Duncan’s multiple range test). Control and H_2_O primed represent unprimed or hydroprimed tomato seedlings. C-PC, *T. viride, T. asperellum, B. bassiana* represent tomato seedlings bioprimed individually with C-PC, *T. viride, T. asperellum, B. bassiana* biostimulants; respectively. *FW* Fresh weight, *DW* Dry weight

Similarly, the enhancement in both germination and growth indices of tomato seedlings primed with C-PC was in agreement with Muñoz-Rojas et al. [45] and Chua et al. [46] that the biopriming of *Eucalyptus gamophylla, Senna notabilis* and *Acacia hilliana* seeds with *Microcoleus sp* and *Nostoc sp* produced seedlings with a longer shoot and root lengths. Also, Haroun and Hussein [47] demonstrated an increase in growth indicators in *Lupius termis* treated with *Cylindrospermum muscicola* and *Anabaena oryzae* extracts. Essa et al. [48] recorded an elevation in the seed germination and the seedling growth criteria of *Sorghum durra* with *Anabaena oryzae* and *Synechococcus* sp. This elevation could be attributed to cyanobacteria’s bioactive compounds, minerals and trace elements, which have the ability to enhance the phytohormones levels and play a crucial role in plant growth regulation, metabolism, and development [46, 49].

### Effects of seed biopriming on primary metabolites

**Fig. 6:**
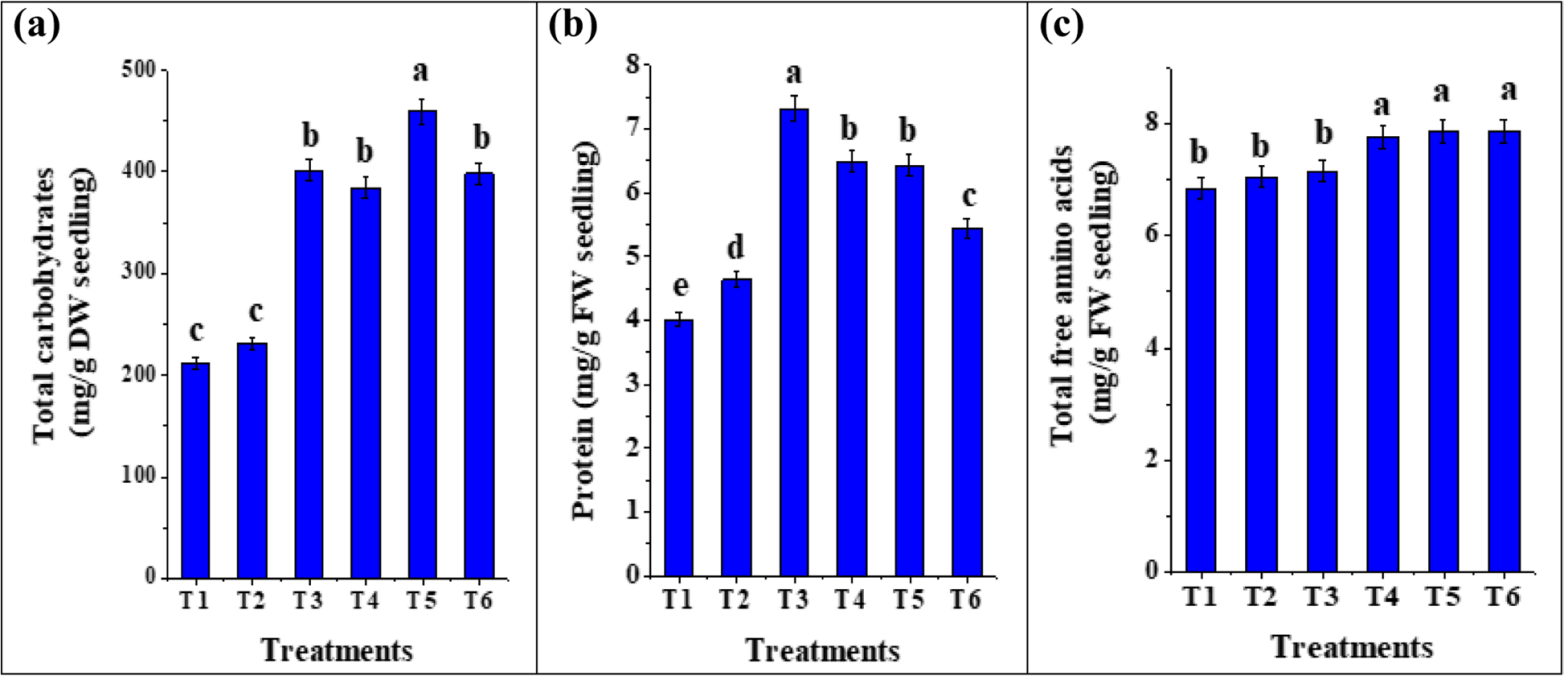
Contents of primary metabolites (total carbohydrates, protein and total free amino acids) in tomato seedlings bioprimed with C-PC, *T. viride*, *T. asperellum* and *B. bassiana* after 12 days of growth. (T1) unprimed *S. lycopersicum* seedlings (T2) hydroprimed *S. lycopersicum* seedlings (T3) *S. lycopersicum* bioprimed with C-PC (T4) *S. lycopersicum* bioprimed with *T. asperellum* (T5) *S. lycopersicum* bioprimed with *T. viride* (T6) *S. lycopersicum* bioprimed with *B. bassiana*. FW: Fresh weight; DW: Dry weight. *Values are means ± SE (Standard Error). Bars labeled with the different alphabet(s) are significantly different (Duncan’s Multiple Range Test, *p* < 0.05)

Changes in the accumulation of the biomolecules show the real impact of treatments in the plants. To assess the effects of C-PC, *T. viride*, *T. asperellum* and *B. bassiana* on the accumulation of the primary metabolites, we measured total carbohydrates, protein and TFAA contents in tomato seedlings (Fig. 6a-c). Also, Pearson’s correlation was analyzed to demonstrate the relation between growth indices and primary metabolites; there were significant positive correlations between the seedling DW with protein (*r* = 0.856), carbohydrates (*r* = 0.825), and TFAA (*r* =0.766) (Table 3). From the quantitative estimation of Fig. 6, there were significantly (*p* < 0.05) higher amounts of total carbohydrates and TFAA contents in tomato seedlings bioprimed with *T. viride* (459.32 mg/g DW and 7.87 mg/g FW; respectively), followed by seedlings bioprimed with C-PC (401.31 mg/g DW and 7.14 mg/g FW) and *B. bassiana* (398.17 mg/g DW and 7.87 mg/g FW).

**Table 3:** Pearson’s correlation matrix between growth indices (shoot height, radicle length, seedling length, seedling fresh weight (FW) and seedling dry weight (DW)) and some biochemical parameters (protein, carbohydrates, TFAA (total free amino acids), amylase, protease, flavonoids, phenolics and shikimic acid)

Measured parameters	Shoot height	Radicle length	Seedling length	Seedling FW	Seedling DW	Protein	Carbohydrates	TFAA	Amylase	Protease	Flavonoids	Phenolics	Shikimic acid
Shoot height	1	0.235	0.965^a^	0.736^a^	0.785^a^	0.788^a^	0.933^a^	0.716^a^	0.815^a^	0.799^a^	0.921^a^	0.680^a^	0.748^a^
Radicle length		1	0.444	0.239	0.357	0.213	0.175	0.219	0.142	0.361	0.193	0.314	−0.033
Seedling length			1	0.673^a^	0.792^a^	0.763^a^	0.887^a^	0.680^a^	0.771^a^	0.807^a^	0.878^a^	0.688^a^	0.657^a^
Seedling FW				1	0.661^a^	0.597^a^	0.690^a^	0.651^a^	0.578^b^	0.684^a^	0.709^a^	0.621^a^	0.552^b^
Seedling DW					1	0.856^a^	0.825^a^	0.766^a^	0.875^a^	0.973^a^	0.869^a^	0.593^a^	0.509^b^
Protein						1	0.873^a^	0.532^b^	0.918^a^	0.863^a^	0.815^a^	0.673^a^	0.481^b^
Carbohydrates							1	0.757^a^	0.908^a^	0.800^a^	0.944^a^	0.691^a^	0.809^a^
TFAA								1	0.631^a^	0.728^a^	0.824^a^	0.579^b^	0.820^a^
Amylase									1	0.843^a^	0.906^a^	0.44	0.649^a^
Protease										1	0.880^a^	0.628^a^	0.483^b^
Flavonoids											1	0.571^b^	0.820^a^
Phenolics												1	0.424
Shikimic acid													1

^a^ Correlation is significant at the 0.01 level (2-tailed)

^b^ Correlation is significant at the 0.05 level (2-tailed)

These results may highlight the promoting effect of fungal endophytes on the contents of these primary metabolites. Also, the results showed that the level of amino acids increased with a parallel increase in protease activity (Fig. 8) in all biopriming treatments. As, amino acids are derived from the degradation of intracellular proteins and their amount in plant tissues are carefully regulated to just meet the requirements for the biosynthesis of proteins [50].

Our findings of increasing the protein and carbohydrates contents are in agreement with Singh et al. [51] who recorded a significantly higher amount of total soluble sugar and proteins in the roots and leaves of *Zea mays* plants bioprimed with *Pseudomonas aeruginosa*. The enhancement of protein, TFAA and carbohydrates contents was also evident from increased plant growth parameters (Table 2). Macuphe et al. [44] revealed a significant increase in protein content of lettuce plants following endophyte colonization which is involved in carbohydrate metabolism, defense and photosynthesis [52, 53]. Since, proteins play an important role in the growth and nutritive value of plants and can mediate the production of antioxidants [54]. Also, White and Torres [55] reported that plants colonized by endophytes produce more glucose and fructose.

Moreover, the C-PC application to tomato seeds has a positive effect on the protein (7.31 mg/g FW) (Fig. 6b), total carbohydrates (401.31 mg/g DW) (Fig. 6a) and TFAA levels (7.14 mg/g FW) (Fig. 6c). Our results were in coherence with Osman et al. [20] that *Spirulina* suspension can increase the creation of proteins and amino acids in roots and shoots of faba bean. Also, Haroun and Hussein [47] demonstrated an increase in nitrogenous chemical content and carbohydrates in shoots of *L. termis* treated with *C. muscicola* and *Anabaena oryzae* extracts. Mógor et al. [56] found that applying *S. platensis* lyophilized biomass, high in L-amino acids, stimulated red beet carbon metabolism and sugar content. Yakhin et al. [57] and Ana [58] documented that cyanobacteria release various kinds of biologically active substances like proteins, vitamins, carbohydrates, amino acids, polysaccharides and phytohormones, which function as signalling molecules to promote plant growth. Also, Nawrocka et al. [22] and Mógor et al. [56] found that the most essential amino acids, polyphenols, and vitamins such as tocopherol and ascorbic acid are abundant in C-PC from *Spirulina* extracts.

#### Effect of biopriming on secondary metabolites in tomato seedlings

**Fig. 7:**
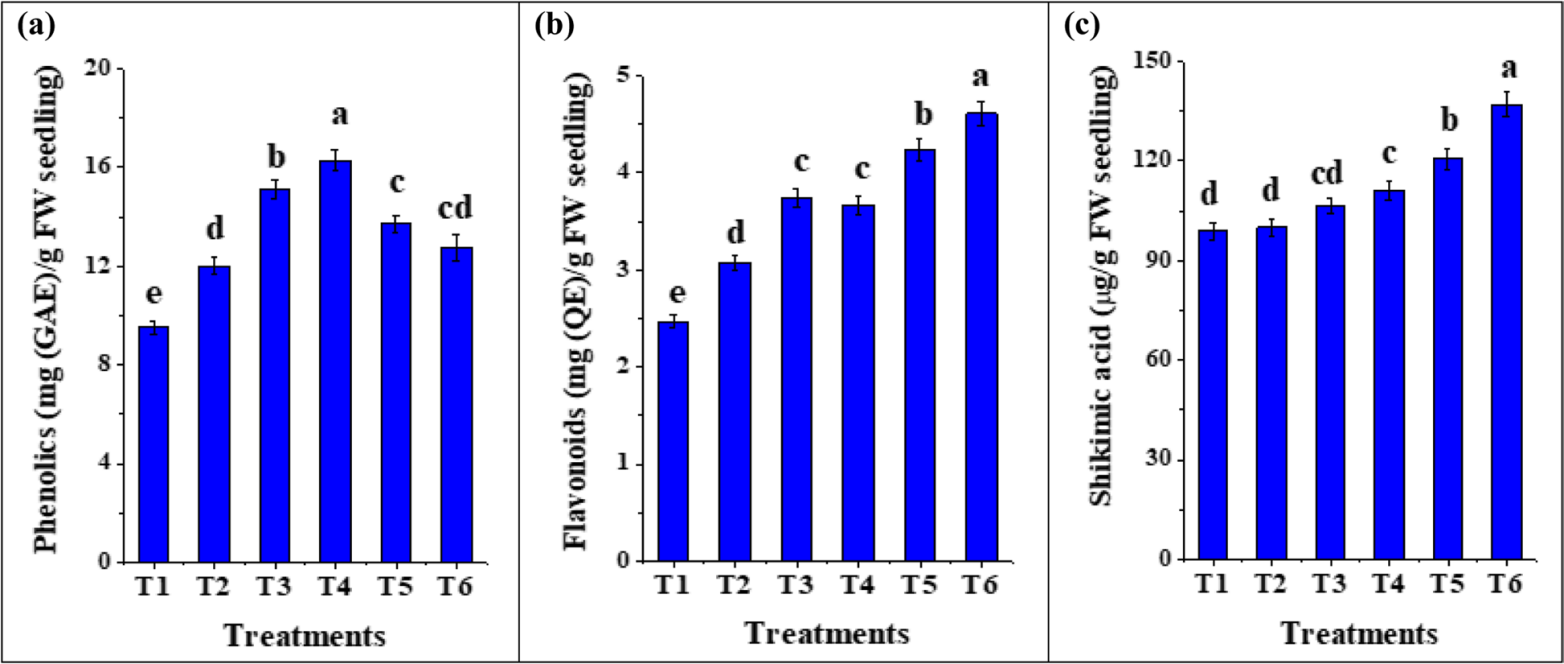
Contents of secondary metabolites (phenolics, flavonoids and shikimic acid) in tomato seedlings bioprimed with C-PC, *T. viride*, *T. asperellum* and *B. bassiana* after 12 days of growth. (T1) unprimed *S. lycopersicum* seedlings (T2) hydroprimed *S. lycopersicum* seedlings (T3) *S. lycopersicum* bioprimed with C-PC (T4) *S. lycopersicum* bioprimed with *T. asperellum* (T5) *S. lycopersicum* bioprimed with *T. viride* (T6) *S. lycopersicum* bioprimed with *B. bassiana*. FW: Fresh weight; DW: Dry weight; GAE: Gallic acid equivalent; QE: Quercetin equivalent. *Values are means ± SE (Standard Error). Bars labeled with the different alphabet(s) are significantly different (Duncan’s Multiple Range Test, *p* < 0.05)

Phenolic and flavonoid compounds are essential for plant functions due to their participation in defensive systems, plant tolerance to a variety of biotic and abiotic stresses, growth and development [59–61]. As well, shikimic acid is the precursor to a wide variety of secondary metabolites that play a key role in plant defense mechanisms [62]. We measured the total phenolic, flavonoids and shikimic acid contents in tomato seedlings bioprimed with C-PC, *T. viride*, *T. asperellum* and *B. bassiana*. Our results (Fig. 7a-c) revealed a significant (*p* < 0.05) increase in their contents with priming. Also, in hydroprimed tomato seedlings (12.01 mg/g seedling FW), a significant (*p* < 0.05) effect on phenolic content was detected compared to unprimed ones (9.5 mg/g seedling FW) (Fig. 7b). Moreover, the highest shikimic acid and flavonoids contents were detected in seedlings primed with *B. bassiana*. These results may highlight the promoting effect of fungal endophytes on increasing these secondary metabolites contents in tomato seedlings. In agreement with our results, the aforementioned studies reported an increase in the accumulation of total flavonoids and total phenolic compounds following endophyte colonization in maize seedlings and tomato plants [52, 63]. Singh et al. [64] also reported an upsurge in shikimic acid content in chickpea leaves treated with triple microbe consortium. There are many plausible explanations for the higher production of secondary metabolites (alkaloids, terpenoids, flavonoids, and phenols). Perhaps maybe it is due to their direct production by the endophyte or the endophyte assists indirectly by influencing their production on the host plant [63]. Moreover, Zaprometov and Nikolaeva [65] and Kovaleva et al. [66] demonstrated the role of polyphenols in regulating plant growth and development, since they affect the biosynthesis of indol-3-acetic acid which plays a key role in both root and shoot development. They also play an important role in the metabolism of plant cells, affecting different physiological processes such as cell division and expansion, membrane permeability, nutrient uptake, enzymatic activity and respiration [67]. However, Moloinyane and Nchu [68] documented that *B. bassiana* did not have any significant effect on total polyphenol, alkaloid, and flavonoids in Grapevine plants. In another study, the total phenolic content of 15-day-old cotton seedlings was reported to be higher than that of 5-day-old seedlings after *Cladorrhinum foecundissimum* colonization [69]. Also, an augmentation in their contents in tomato seedlings primed with C-PC extract was detected, Goiris et al. [70] linked phenolic compounds found in microalgae and cyanobacteria to antioxidant properties, and they play a significant role in growth, reproduction, and stress tolerance. Therefore, C-PC, *T. viride*, *T. asperellum* and *B. bassiana* not only promoted plant growth but also stimulated the accumulation of shikimic acid, phenolic and flavonoid contents in bioprimed seedlings.

#### Effects of seed biopriming on hydrolytic enzymes

**Fig. 8:**
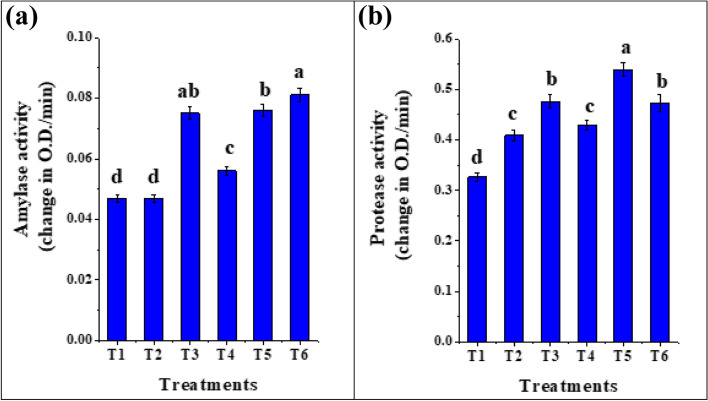
Hydrolytic enzymes activities (amylase and protease) in tomato seedlings bioprimed with C-PC, *T. viride*, *T. asperellum* and *B. bassiana* after 12 days of growth. (T1) unprimed *S. lycopersicum* seedlings (T2) hydroprimed *S. lycopersicum* seedlings (T3) *S. lycopersicum* bioprimed with C-PC (T4) *S. lycopersicum* bioprimed with *T. asperellum* (T5) *S. lycopersicum* bioprimed with *T. viride* (T6) *S. lycopersicum* bioprimed with *B. bassiana*. *Values are means ± SE (Standard Error). Bars labeled with the different alphabet(s) are significantly different (Duncan’s Multiple Range Test, *p* < 0.05)

During germination, the high molecular weight reserves in the storage organs of the seed are converted into transportable forms and are transported to metabolizing and growing tissues where they are utilized for energy-producing and synthetic events [71]. The major types of these storage reserves in seeds are starch and proteins, where their conversion to transportable forms was accompanied by activation of hydrolytic enzymes such as amylases and proteases. Regarding to Table 3, the Pearson’s correlation showed strong positive correlations between growth indices and hydrolytic enzymes; shoot height exhibited a significant positive correlation with amylase (*r* = 0.815**) and protease (*r* = 0.799**). Our results (Fig. 8a and b) revealed an augmentation in the amylase and protease enzyme activities in tomato seedlings upon biopriming with *T. asperellum*, *T. viride, B. bassiana* and C-PC extract. The highest amylase activity (Fig. 8a) was recorded by *B. bassiana* (0.081 Change in OD/min) followed by seedlings bioprimed with *T. viride* (0.076) and C-PC extract (0.075 Change in OD/min) compared with hydropriming or control ones (0.047 Change in OD/min). This was in line with Robl et al. [72] that endophytic fungi such as *T. atroviride*, *Alternaria* sp., *Annulohypoxylon stigyum* and *Talaromyces wortmannii* are excellent producers of hydrolytic enzymes. These enzymatic activities are essential for providing energy and carbon skeletons to the growing embryo through the respiratory breakdown of utilizable substrates until the seedling becomes photo-synthetically self-sufficient.

Moreover, Caldwell et al. [73] reported the ability of root endophytic fungi, *Philaophora finlandia* and *P. fortinii* to produce hydrolytic enzymes, which were able to break down the major polymeric forms of C, N and P found in plants. Similar results were proved by Marlida et al. [74] and Maria et al. [75]. In this connection, Gholam et al. [76] proved that the enhancement of seed germination by plant growth-promoting fungal inoculants was due to the synthesis of seed germination hormones like gibberellins which triggered the activity of specific enzymes, such as alpha-amylase. Furthermore, Mabood et al. [77] recommended that hydrolytic enzymes are of major interest due to their ability to degrade and lyse pathogen cell wall, and thus they are employed in biocontrol of fungal phytopathogens as Felse and Panda [78] reported in the control of *Sclerotium rolfsii* and *F. oxysporum* through the cell wall degradation by hydrolytic enzymes on beans.

Additionally, the enhancing effect of C-PC extract (Fig. 8) in germinated seedlings of tomato agreed with Osman et al. [79] who recorded an increase in amylases and proteases enzyme activities in germinated seedlings of pea treated with the cyanobacterial extract. Amylases increased the availability of starch assimilation by the hydrolysis of it into glucose [80]. The other groups of enzymes are proteases which play an important role during germination, in the mobilization of stored protein in seed as free amino acids, which are utilized in building necessary protein and enzymes required for the growing embryo [81].

## Conclusion

Seed biopriming is one of the innovative and ecofriendly priming methods, as it is useful not only for enhancing seed germination and seedling vigor, but also for the management of biotic and abiotic stresses. Our recent study tries to fill the gap by examining the comparative effects of these different biostimulants such as *T. viride*, *T. asperellum* and *B. bassiana* and C-PC extract on seed germination, seedling growth and biochemical traits of *Solanum lycopersicum* L. According to our results, the most effective biostimulants were those bioprimed with *T. viride* and *B. bassiana* as compared to other biostimulants (*T. asperellum* and C-PC). Thus, biopriming of seeds with the desired fungal endophytes can be used commercially as an alternative to biofertilizers successfully. Future strategies should apply biopriming with different biostimulants to other plant seeds yet not experimented which will give a better picture of the potential of this technology.

## Methods

### Preparation of priming materials

#### Fungal inoculums preparation

The cultures of *T. viride*, *T. asperellum*, *B. bassiana* were brought from Mycology Lab, Faculty of Science, Zagazig University. The fungal inoculums were prepared from 7-day old culture. In brief, the Petri dish containing the culture was suspended with sterile distilled water. The spores were gently removed using a glass spreader, and then the heterogeneous suspension was filtered using the muslin cloth for removing the mycelial mat. The filtered suspension was diluted with sterile distilled water and adjusted to 10^6^–10^7^ spores/ mL as quantified through the hemocytometer.

#### C-PC crude extract preparation

##### Growth and maintenance of culture

*S. platensis* was obtained kindly from Prof. Dr. Yassin El-Ayouty (Phycology Lab, Faculty of Science, Zagazig University). The culture was maintained in Z-Medium [82] at 28 ± 2 °C under a light intensity of 52–55 μEm^−2^s^− 1^and light/dark cycles of 16:8 h. At mid-logarithmic phase, algal cells were harvested by centrifugation at 10000 rpm (4 °C) for 15 min using Multi-tube under cooling centrifuge (Vision SCIENTIFIC CO., LTD., South Korea), washed three times with sterile distilled water, and air-dried.

##### Extraction and estimation of C-PC

About 10 g of dried algal cells were suspended in 50 mL of Calcium Chloride (10 gL^− 1^). Samples were subjected to freeze-thawing (incubated at − 20 °C until solid, followed by thawing for 24 h at 4 °C in the dark). Phycocyanin was extracted by repeated freezing (− 20 °C) and thawing at room temperature until the blue color becomes visible. Cell debris was removed by centrifugation at 5000 rpm for 10 min using Eppendorf under cooling centrifuge (MIKRO 200R Hettich zentrifugen, Germany) and the extract thus obtained was termed as a crude extract. The amount of C-PC was measured as described by Bennett and Bogard [83] and purity was determined by using the formulae: Purity = A_620_/A_280_. C-PC concentrations (μgPC L^− 1^) were determined according to Lawrenz et al. [84].

##### Purification of C-PC

*Dialysis*

The obtained crude C-PC was dialyzed against the extraction buffer using dialyses membrane (Dialysis membrane-70, MWCO; 12–14 kD) procured from Hi-Media. Dialysis was performed twice against a 1000 mL extraction buffer, first at room temperature and again dialyzed against 1000 mL of extraction buffer at 4 °C overnight. The resultant extract was recovered from the dialysis membrane and filtered through a 0.45 μm filter.

***Ion exchange chromatography***

Phycocyanin further purified by ion-exchange chromatography using a DEAE-Sepharose, from Enzymology and Fungal Biotechnology Lab (EFBL, Faculty of Science, Zagazig University). A column (2 × 30 cm) had been pre-equilibrated with 20 mM sodium acetate buffer containing 50 mM NaCl. After washing with 60 mL of the same buffer, the dialyzed filtered sample was placed on the column; the column was eluted with the same buffer. The elutes were collected in 5 mL fractions. Fractions were collected at a 0.5 mL/min flow rate [85]. Then, the purity of all fractions was checked by equation. The absorption spectrum was also determined by scanning the highly purified sample in the range of 200–800 nm by using UV / VIS Spectrophotometer (T80, PG Instruments Ltd. (UK)).

##### Fourier-Transform InfraRed spectroscopy (FT-IR) spectral analysis

The functional group^’^s profile of the purified phycocyanin from *S. platensis* was done by FT-IR (Bruker, Germany) spectral analysis. The KBr pellet was prepared by mixing 1 mg of the sample with 100 mg of anhydrous potassium bromide. The spectra were recorded from 500 to 4000 cm^− 1^ and 30 scans at a resolution of 4 cm were averaged and referenced against air.

##### Nuclear Magnetic Resonance spectroscopy (^1^H NMR) spectral analysis

The structural feature of the purified phycocyanin from *S. platensis* was evaluated by ^1^H NMR spectra (Bruker, Germany) by following the method of Schanda and Brutscher [86]. Approximately 30 mg of sample was dissolved in 0.5 mL of D_2_O (99.9%) in a NMR tube (5 mm diameter). The ^1^H NMR spectra were taken at 27 °C and the chemical shift was expressed in parts per million (ppm).

### Priming, treatments and experimental conditions

Seeds of tomato (*Solanum lycopersicum* L.; Tomato HYBRID Seven F.1) were gained from the local market of Minia Al-Qamh, El-Sharkia Governorate. For seed biopriming with a spore suspension of *T. viride*, *T. asperellum*, *B. bassiana* and C-PC (*S. platensis* extract), the healthy seeds of tomato were surfaces sterilized with 2% (v/v) sodium hypochlorite (NaOCl) solution for 3 min followed by repeated washing with distilled water and further dried under laminar airflow on autoclaved blotting paper [87]. The surface sterilized and dried seeds were treated by soaking in the spore suspensions of *T. viride*, *T. asperellum*, *B. bassiana* and the extract of C-PC. The control seeds were left un-primed (control) or primed only with sterilized distilled water (hydroprimed). Therefore, the treatments involved were T1: Non primed seeds (Control), T2: Hydropriming, T3: Biopriming with C-PC, T4: Biopriming with *T. viride*, T5: Biopriming with *T. asperellum*, T6: Biopriming with *B. bassiana*. Further, all the seeds were placed in the moist chamber at 98% relative humidity and 25–28 °C and maintained for 24 h [13], after that, they were air dried. Each treatment was replicated 4 times, so, a total of 24 Petri dishes (6*4) were used, each containing 10 seeds. Primed and unprimed tomato seeds were germinated in 9-cm diameter Petri dishes. The dishes were covered with a layer of absorbent cotton and blotter papers and were incubated at 25 ± 1 °C with supplemental day/night lighting of 16/8 h. After 12 days of growth, seedlings were collected from each treatment for measuring germination parameters and the rest seedlings were frozen in liquid nitrogen then immediately grinded in the suitable solvent for each biochemical parameter.

### Bioassay on comparative effects of biopriming with fungal inoculums and C-PC extract on the germination indices and seedling growth

Seeds were considered germinated on radicle visibility (the radicle length was longer than 2.0 mm). After 12 days of growth, seedlings were collected from each treatment for measuring different germination indices and germination parameters to indicate the influence of different biostimulants. Germination percentage (%) [88], germination index (GI) [89], seedling length vigor index (SLVI) [90] and seedling weight vigor index (SWVI) [91] are examples of the germination indices.

$$Germination\%= Total\ number\ of\ germinated\ seeds/ Total\ seeds\ sown\ X\ 100$$

$$GI= Total\ number\ of\ germinated\ seeds/ Total\ number\ of\ days$$

$$SLVI=\left[ seedling\ length\ (cm)\times seed\ germination\ \left(\%\right)\right]$$

$$SWVI=\left[ seedling\ DW\ (mg)\times seed\ germination\ \left(\%\right)\right].$$

After that, seedlings were harvested, and readings were taken regarding seedling growth according to ISTA protocols [92]. Shoot height and radical length was measured in 5 normal seedlings randomly obtained. The seedling fresh weight (FW) and dry weight (DW) were recorded after oven drying at 60 °C for 48 h.

### Bioassay on comparative effects of biopriming with fungal inoculums and C-PC extract on tomato seedlings primary metabolites contents

#### The total protein content

The fresh seedlings of known weight (1 g) were ground in a mortar and pestle using 50 mM phosphate buffer (pH 7). The resultant homogeneous solution was centrifuged at 8000 rpm for 15 min at 4 °C. Supernatant constituting the crude extract of amylase and protease was collected and aliquots were used for protein content and hydrolytic enzymes activity estimation. The protein content of tomato seedlings from each treatment was calculated [93] with some modifications. The mixture was again subjected to shaking for 10 mins after adding alkaline copper sulfate reagent and Folin’s reagent. The whole mixture was placed in an incubator for 30 min. The absorbance of each sample was recorded at 700 nm against blank. The concentration of total soluble proteins was determined with the reference curve of bovine serum albumin as a standard.

#### The total carbohydrates

A known tomato seedlings dry weight of all treatments were separately hydrolyzed in boiling water for 3 h with 10 mL 2.5 N HCl and then cooled. It was further neutralized with sodium carbonate and then centrifuged at 5000 rpm for 15 min and the supernatant (0.1 mL) was used for total carbohydrates estimation by phenol sulphuric acid method [94]. The 2.5 mL of H_2_SO_4_ was added to the reaction mixtures and subjected to vigorous stirring followed by recording the absorbance at 490 nm. The amount of carbohydrates (mg/g DW) was calculated using the glucose standard curve.

#### Total free amino acids (TFAA)

A known seedlings fresh weight of tomato were extracted in 5 mL of 80% ethanol and centrifuged at 6000 rpm for 30 min to measure their TFAA contents by Yemm et al. [95]. The test extract was taken and TFAA was estimated using ninhydrin reagents containing 1% ninhydrin in 0.5 M citrate buffer, pH 5.5, glycerol (87%) and 0.5 M citrate buffer pH 5.5 in the ratio of 5:12:2. After vigorous shaking contents were heated in a boiling water bath for 10 mins and after cooling, absorbance was measured at 570 nm with ethanol serving as blank in place of test extract. Absorbance readings were converted to mg amino acid g^− 1^ fresh weight of seedling using a glycine standard curve.

### Bioassay on comparative effects of biopriming with fungal inoculums and C-PC extract on tomato seedlings secondary metabolites contents

#### The total phenolic content

The total phenolic content of the tomato seedlings was assessed from the seedling extract [96] after 95% ethanol extraction. According to the protocol, around 200 μL of the prepared extract was poured into a test tube with 1.4 mL of distilled water and 0.1 mL of 50% Folin-Ciocalteu phenol reagent. The sample was left for 3 mins and then sodium carbonate (0.4%) was added. The resultant mixture was kept for 2 h and then subjected to a gentle vortex. The absorbance was recorded at 650 nm. The gallic acid was used as a standard against which the total phenolic content was measured and expressed as mg/g FW of gallic acid equivalent (GAE).

#### The polyphenols (flavonoid and shikimic acid) content

The total flavonoid content of the tomato seedlings from different treatments was measured by following the AlCl_3_ colorimetric assay described by Zou et al. [97] using quercetin standard curve and was expressed as μg/g FW of quercetin equivalent (QE). Seedlings were taken and cut into very small pieces; 95% ethanol was added to make a fine paste. This mixture turned into a suspension and was subjected to centrifugation for 10 mins. The supernatant (200 μL) was collected and poured into a volumetric flask and around 5 mL of distilled water was added followed by the addition of 0.7 mL of 5% NaNO_3_ and 0.6 mL of 10% AlCl_3_. The resultant solution was put to rest for 5 mins. The solution was again left for 1 min after adding 3 mL of 1 M NaOH and 2.5 mL of distilled water and mixed thoroughly. The absorbance was recorded at 510 nm using a spectrophotometer versus a blank.

A known seedling fresh weight of tomato seedlings was ground in 2 mL 0.25 M HCl and then centrifuged for 30 min for determination of shikimic acid concentration according to Zelaya et al. [98] using the shikimic acid standard curve. The supernatant (50 μL) reacted with 0.5 mL of a 1% periodic acid and incubated at room temperature for 3 h. After incubation, 0.5 mL 1 M NaOH and 0.3 mL 0.1 M glycine were added. The absorbance was measured at 380 nm.

### Determination of hydrolytic enzymes (Amylase and protease)

The supernatant obtained from homogenizing 1 g fresh seedlings with 50 mM phosphate buffer (pH 7) was used to estimate the hydrolytic enzymes activity. The amount of starch hydrolyzed by the action of amylases was measured according to Johnson [99]. Protease activity was measured in an azocasein assay [100]. Specific enzyme activity was expressed as change in optical density min^− 1^.

#### Statistical analysis, correlation analysis and figure preparation

The experimental design used in this study was carried out in a completely randomized design of 6 seedlings per treatment and each treatment was repeated in four sets. The obtained experimental data were processed by the mathematical and statistical methods using the SPSS software (version 15) statistical package. Descriptive statistics were used to process the obtained data which were expressed as mean ± Standard Error (SE). Comparison of mean values of all primed and non-primed samples were done using a One Way ANOVA test and Duncan’s test at *p* < 0.05. Pearson’s correlation coefficients (*r*) were carried out to understand the relationship between growth indices and different biochemical parameters using SPSS. Figures were assembled using OriginPro 8.5 for data analysis and graphing software.

## Data Availability

All data generated or analyzed during this study are included in this published article.

## Abbreviations

C-PC: C-phycocyanin
DW: Dry weight
FW: Fresh weight
GAE: Gallic acid equivalent
GI: Germination Index
SE: Standard Error
SLVI: Seedling length vigor index
SWVI: Seedling weight vigor index
TFAA: Total free amino acids
QE: Quercetin equivalent
